# Significant Reduction of Systematic PAH and NPAH Discharges from Hazardous Waste Treatment by the Advanced Scrubbing and Circular GASMILD Combustion

**DOI:** 10.1021/acsomega.5c01462

**Published:** 2025-09-26

**Authors:** Tang Wei, Hsieh Yu-Lun, Lin Sheng-Lun, Fang Guor-Cheng, Lee Hyojun

**Affiliations:** † School of Mechanical Engineering, Beijing Institute of Technology, Beijing 100081, China; ‡ Department of Environmental Engineering, National Cheng Kung University, Tainan 70101, Taiwan; § Department of Safety, Health, and Environmental Engineering, Hungkuang University, Taichung 43302, Taiwan; ∥ Department of Nuclear Engineering, Hanyang University, SeongDong-Gu, Seoul 04763, Korea

## Abstract

This study systematically investigates the application of sludge and fly ash circular combustion (SFACC) under gasification-moderate or intense low-oxygen dilution (GASMILD) incineration in a hazardous waste thermal-treatment system (HAWTTS) to achieve significant reductions in hazardous air pollutants. The aim of this research is to evaluate whether integrating residue reinjection into a closed-loop GASMILD system can enhance the net removal efficiency of polycyclic aromatic hydrocarbons (PAHs) and nitro-PAHs (NPAHs), while revealing critical insights into pollutant behavior and control. The research demonstrates that while conventional incineration effectively destroys a substantial portion of polycyclic aromatic hydrocarbons (PAHs, 98.7% destruction) and nitro-PAHs (NPAHs, 89.2% destruction), and air pollution control devices (APCDs) further reduce their emissions (63.9% and 88.1% reduction, respectively), pollutant migration and concentration in sludge and fly ash significantly diminish overall system removal efficiency (6.1–43.2%). Crucially, implementing SFACC achieved a closed-loop system, demonstrably enhancing the net reduction of PAHs and NPAHs to 99.2% and 93.7%, respectively, by eliminating solid residue discharge. Detailed particle size distribution analysis in the stack flue gas provided valuable insights into respiratory health risks. The investigation discovered that their absolute emission levels remained low while SFACC increased particulate matter concentrations and the relative content of more toxic, higher molecular-weight PAHs and NPAHs. These findings provide strong scientific evidence supporting the use of SFACC under GASMILD incineration as a novel and effective approach to achieving comprehensive, system-wide control of hazardous air pollutants from hazardous waste incineration.

## Graphical abstract

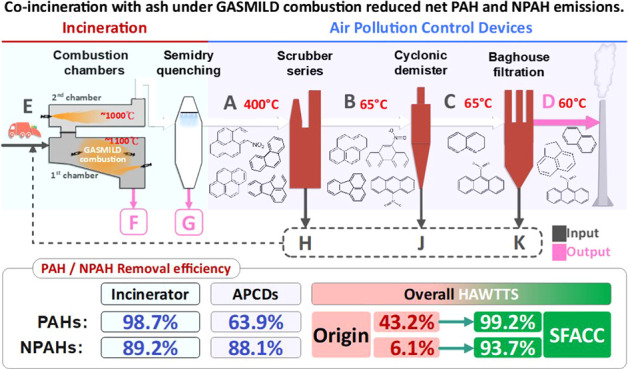

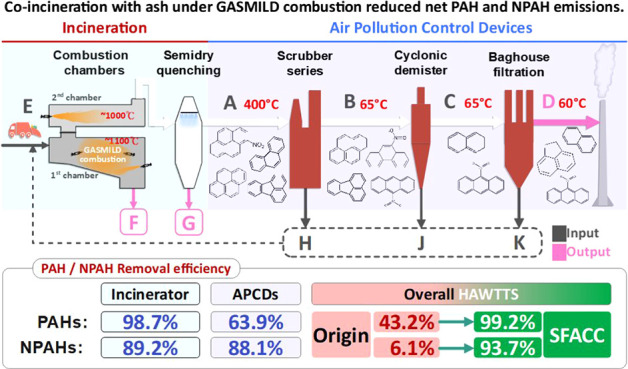

## 1 Introduction

Waste incineration is a critical component of waste management, but unstable combustion and complex feedstock compositions often lead to the formation of undesirable byproducts.^1^ Recent studies have elucidated that the formation of polycyclic aromatic hydrocarbons (PAHs) and nitro-polycyclic aromatic hydrocarbons (NPAHs) in waste incineration primarily involves several competing mechanisms. At high temperatures, incomplete combustion of organic matter leads to the survival of aromatic precursors, which can subsequently undergo hydrogen abstraction–acetylene addition reactions, radical recombination, and surface-catalyzed polymerization during flue gas cooling.^2^ Conversely, the thermal destruction of PAHs is mainly governed by high-temperature pyrolysis and radical oxidation processes, with hydroxyl (OH•) and atomic oxygen (O•) radicals playing dominant roles.^3^ While air pollution control devices (APCDs) are used to mitigate these emissions, they often generate substantial quantities of toxic residues, creating a secondary pollution problem. Carcinogenic PAHs and NPAHs in SFA highlight potential environmental and human health risks, particularly through soil and atmospheric pathways. Enhanced APCD efficiency, stricter feedstock control, and post-treatment stabilization of residues are essential to mitigate these risks.

A promising strategy to address secondary pollution in hazardous waste thermal treatment systems (HAWTTS) involves reintegrating toxic residues into the waste feedstock.^4^ Prior research on coincineration of sewage sludge and municipal solid waste has shown alterations in incineration dynamics and heavy metal behavior, notably enhancing copper stability^5^ Ash, a major component of these residues, significantly influences combustion through radiative heat transfer, surface reactions, and gas–solid interactions. Its high specific surface area and porous structure provide abundant active sites for the adsorption of semivolatile organic compounds (SVOCs), particularly facilitating the capture of less volatile, high molecular-weight PAHs and NPAHs.^6^ Additionally, metallic species within the ash can promote heterogeneous reactions that influence the formation and decomposition pathways of these pollutants.^7^ While ash can improve combustion stability and efficiency,^8^ excessive deposition can lead to operational issues.^9^ Critically, ash’s high surface area and porosity provide adsorption sites for SVOCs, potentially facilitating the removal of gaseous PAHs and NPAHs.^10^ However, the specific impacts of ash addition to feedstock on PAH and NPAH formation, emission characteristics, and particle size distribution (PSD) remain poorly understood, necessitating further investigation.

MILD combustion technology offers a promising solution by achieving a uniform distribution of temperature and chemical species, thereby minimizing local fluctuations that commonly lead to incomplete combustion, particulate matter (PM), and SVOC emissions in conventional diffusion combustion.^11^ MILD combustion’s highly diluted and preheated environment promotes distributed ignition and stable flame propagation, effectively mitigating the detrimental effects of high moisture content, which typically exacerbates incomplete combustion under diffusion-controlled conditions.^12^ In previous studies, MILD combustion was operated at a typical flue gas temperature of approximately 1100 °C for waste incineration. These conditions facilitate stable combustion, suppress PAHs and NO_*x*_ formation, and limit the generation of incomplete combustion byproducts.^13,14^ While recent research demonstrates MILD combustion’s effectiveness in treating solid waste blends, achieving high burnout and reducing NOx and PM_2.5_,^15^ the impact of fly ash addition on PM mass concentration and the subsequent implications for APCD performance require careful consideration. This presents a knowledge gap regarding the application of MILD combustion to heterogeneous waste streams containing recycled residues.

Common APCDs like activated carbon injection, baghouse (BH) filters, wet/dry electrostatic precipitators (ESPs), and scrubbers (SCBs) are used for PM and SVOC removal.^16,17^ Although effective, these technologies have drawbacks, including high costs, potential carbon emissions increases, and the “memory effect” in BHs.^16,18,19^ The removal mechanisms of APCDs include physical adsorption, electrostatic attraction, mechanical interception, and gas–liquid absorption, with their performance influenced by temperature, humidity, particle properties, and device-specific operating conditions.^20^ However, the specific mechanisms governing APCD removal efficiency under conditions of increased PM mass concentration and altered PSD, specifically resulting from residue recirculation in a MILD combustion environment, remain largely unknown. This constitutes a significant research gap.

These knowledge gaps include the limited understanding of how recycled residues such as sludge and fly ash affect pollutant formation and removal efficiency under MILD combustion, as well as the insufficient exploration of APCD performance under altered particle loading and composition. To address these issues, this study investigates an innovative, full-scale HAWTTS employing a novel combustion mode combining gasification and flameless combustion, achieved through optimized incinerator flow field design. By reintegrating APCD-generated residues (sludge from the SCB and cyclone demister and fly ash from the BH) into the waste feedstock, this research evaluates the effectiveness of a closed-loop system in achieving net reductions in pollutant discharge—a crucial step toward sustainable waste incineration. Comprehensive sampling and analysis at key HAWTTS locations enabled detailed characterization of emission concentrations, PM particle size distributions, and PAH/NPAH congener mass and toxicity concentrations. This data allows for a novel investigation into the effects of fly ash blending on PM, PAH, and NPAH emissions under flameless combustion conditions, and a critical assessment of how high fly ash concentrations impact the removal mechanisms of different APCD units. The findings provide crucial scientific insights into the complex interplay between residue recirculation, MILD combustion, and APCD performance, contributing to developing more effective and sustainable hazardous waste incineration strategies.

## 2 Materials and Methods

### 2.1 Hazardous Waste Thermal Treatment System (HAWTTS)

The experiments were conducted using the HAWTTS located at the Environmental Resource Management Research Center of National Cheng Kung University, Taiwan. This facility comprises a primary combustion chamber, a secondary combustion chamber, and a series of APCDs (see Figure 1). The incinerator has an annual treatment capacity of 1,200 tons, with a capacity of 800 tons for organic waste and 400 tons for inorganic waste. The waste feedstock consisted of discarded materials generated from laboratory operations, the detailed composition and properties of which are provided in Table S1. Moisture content was measured using a moisture analyzer (Kyoto Electronics Manufacturing Co., Ltd., MKV-710B). The lower heating value and density were determined using a bomb calorimeter (IKA C3000) and a digital densitometer (METTLER TOLEDO Densito), respectively. The elemental contents of metals, sulfur, and chlorine were analyzed using an X-ray fluorescence (XRF) spectrometer (Olympus Innov-X DS-4050).

**Figure 1:**
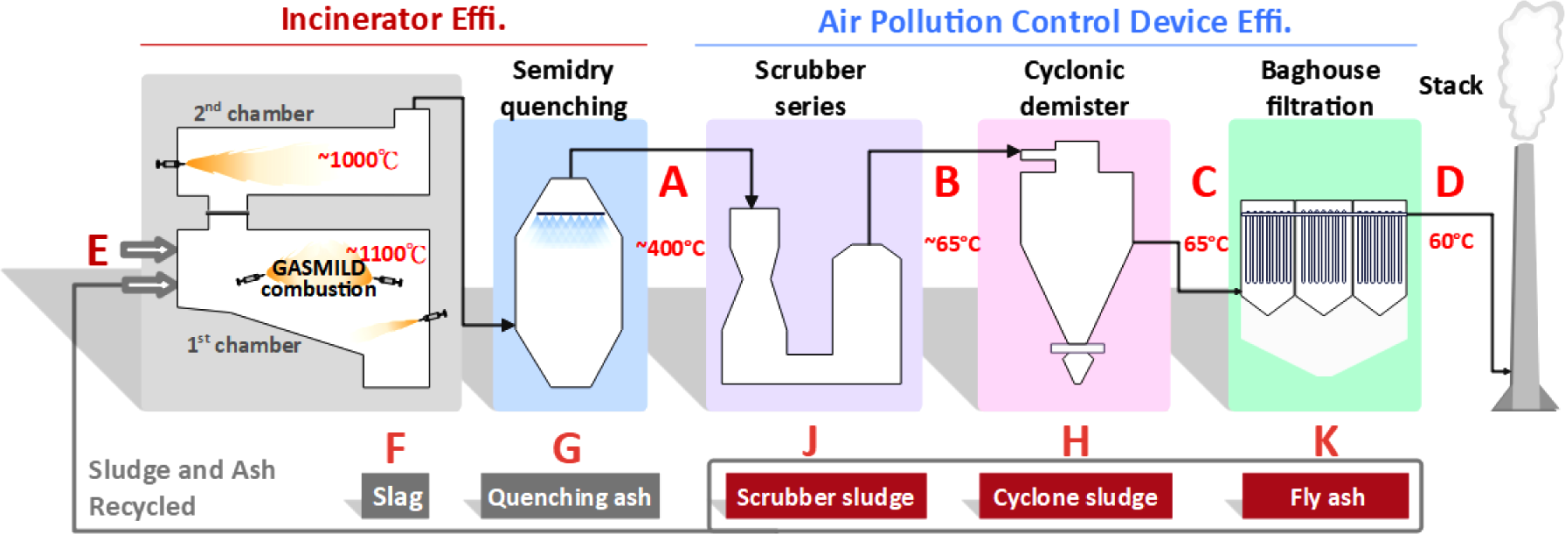
Schematic diagram of HAWTTS.

The operational parameters and combustion conditions of the primary and secondary combustion chambers are summarized in Table S2. The system was operated with a mass flow rate of 100 kg/h for solid hazardous waste and 120 kg/h for liquid waste. The liquid waste stream primarily consisted of industrial wastewater with isopropanol as the major component.

#### 2.1.1 GASMILD Combustion

This study employed a GASMILD (gasification moderate or intense low-oxygen dilution) combustion strategy, a thermal treatment method integrating gasification with flameless combustion. By adjusting the position, angle, equivalence ratio, and flow rate of the injectors, a swirl field was established within the incinerator to achieve flameless combustion. Upon entering the primary combustion chamber, the waste undergoes pyrolysis and gasification in an oxygen-lean environment without direct contact with auxiliary fuel. The energy required for these endothermic reactions is supplied by the MILD combustion occurring in the main combustion zone. In this environment, pyrolysis first generates volatile gases and char, while gasification is sustained through heterogeneous reactions between char and gaseous agents such as CO_2_ and H_2_O. Notably, the water–gas shift reaction (CO + H_2_O ⇌ CO_2_ + H_2_) plays a pivotal role in regulating the local composition of the gas phase by enhancing the generation of H_2_ and CO_2_, thereby maintaining a reducing atmosphere favorable for continuous gasification.^21^ The resulting products (H_2_O, CO, CO_2_, and hydrocarbons) and heat are circulated within the combustion chamber by a carefully designed swirl field, called IFF in the previous study.^13^

MILD combustion of combustible gases is achieved within the primary combustion chamber under the control of three injectors. Key injector parameters include jet velocity, angle, and fuel-to-air ratio, which are precisely regulated to generate a strong turbulent swirl field that promotes MILD combustion.^13^ Real-time monitoring of oxygen and temperature ensures stable combustion conditions, maintaining a low-oxygen environment (<3% O_2_) that suppresses localized high-temperature zones and reduces the formation of thermal NO_*x*_. Meanwhile, the distributed combustion regime under low-oxygen conditions also influences the pyrolysis and oxidation pathways of organic compounds, mitigating the formation of incomplete combustion products such as PAHs and NPAHs. Thorough gas mixing and a uniform temperature distribution (approximately 1100 °C). High-temperature vitrification of inorganic materials occurs near the bottom ash outlet of the primary combustion chamber. Combustion products from the primary chamber are then transferred to the secondary combustion chamber for further oxidation under oxygen-rich conditions. A more detailed description of the HAWTTS can be found in previous studies.^13^

#### 2.1.2 Modified Air Pollution Control System

The APCDs in this HAWTTS consist of a semidry cooling tower, a wet scrubber (SCB), a cyclone separator (CYCD), and a baghouse (BH) filter equipped with a powdered activated carbon injection system (see Figure 1). The SCB is designed to remove acidic pollutants, particulate matter (PM), and volatile organic compounds from the flue gas. It operates with an alkaline liquid injection rate of 211.9 L/min at a pH of 9.6. This study, the SCB also demonstrated efficacy in removing polychlorinated dibenzo-p-dioxins and dibenzofurans (PCDD/Fs) through temperature control.^22^ The CYCD primarily removes water mist from the flue gas exiting the SCB, protects the BH filter, and contributes to PM removal. Final polishing of the flue gas is achieved by the BH filter in conjunction with activated carbon injection. The injected activated carbon has a particle diameter of less than 75 μm, a moisture content below 3%, and an apparent density between 0.4 and 0.5 g/cm^3^. The sludge generated by the SCB and CYCD, and the fly ash collected by the BH, are considered secondary pollutants and are the focus of the residue reintegration strategy investigated in this study.

### 2.2 Experimental Design

To assess the impact of residue reintegration on emissions, sampling points were strategically located within each component of the HAWTTS, as illustrated in Figure 1. Point E represents the feed inlet to the primary combustion chamber. In contrast, Point A represents the flue gas outlet of the secondary combustion chamber. Points B, C, and D correspond to the SCB, CYCD, and stack flue gas outlets, respectively. In addition to flue gas sampling, sampling points were established to collect residues generated by the incinerator and APCDs. Point F corresponds to the bottom ash outlet of the incinerator, Point G to the sludge collected from the quenching tower, Point H to the sludge from the SCB, Point J to the sludge from the CYCD, and Point K to the fly ash collected by the BH. In addition, detailed descriptions of all sampling locations, their operating conditions, and potential sources of interference have been provided in the Supporting Information (Table S4). The sensitivity analysis of operational conditions based on CO and NO emissions was conducted using computational fluid dynamics simulations, and the results are provided in Table S5, which can be found in the Supporting Information.

Previous studies have shown that residues generated from APCDs, such as the sludge from the SCB and CYCD and the fly ash from the BH, can contain high concentrations of toxic compounds, particularly polychlorinated dibenzo-p-dioxins and dibenzofurans (PCDD/Fs).^13^ Therefore, this study selected sampling points at these critical locations to target the residues most likely to accumulate hazardous substances specifically. The experimental design involved collecting and reintegrating APCD-generated residues, specifically sludge from the SCB and CYCD, and fly ash from the BH (collectively referred to as SFA). The collected SFA was dewatered to form filter cakes, which were then blended with the primary waste feedstock and coincinerated by GASMILD operation, called sludge and fly ash circular combustion (SFACC). The SFA generation rates were as follows: Point F (4.0 kg/h), Point G (1.6 kg/h), Point H (22.5 kg/h), Point J (0.5 kg/h), and Point K (1.6 kg/h). After dewatering, the total mass of the recycled filter cakes constituted 15% of the total mass of the waste feed, equivalent to a feeding rate of 15.0 kg/h. The economic analysis indicates that, after accounting for additional operational, labor, and maintenance costs, the gasification-MILD combustion system with ash-sludge recirculation achieves net savings of approximately 903 USD per ton of waste treated, demonstrating cost-effectiveness.

### 2.3 Sampling and Analytical Procedures for PAHs, NPAHs, and PM

#### 2.3.1 PAH and NPAH Congeners and Toxicity Equivalency Factors

The PAH family comprises numerous compounds, ranging from low molecular weight (LMW) PAHs such as naphthalene (NaP), acenaphthylene (AcPy), and acenaphthene (AcP) to high molecular weight (HMW) PAHs such as fluoranthene (FL), pyrene (Pyr), and benzo­[a]­anthracene (BaA).^23,24^ HMW PAHs are of particular concern due to their strong carcinogenic properties and persistence in the environment.^24^ In this study, 16 PAH compounds were selected as representative indicators of combustion-related pollution based on the following criteria: (1) their high frequency of detection in emissions from waste incineration and combustion processes; (2) their toxicological relevance, including carcinogenicity and mutagenicity; and (3) their inclusion in widely recognized regulatory frameworks and environmental monitoring programs, such as the U.S. EPA priority pollutant list. These selected PAHs cover a range of molecular weights and physicochemical properties, allowing a comprehensive assessment of emission characteristics. The full names and corresponding abbreviations of these PAHs are provided in Table S3.

Furthermore, NPAHs encompass a diverse group of compounds characterized by nitro groups attached to the aromatic rings of PAHs.^25^ This study included a selection of NPAHs as representative indicators of combustion-related pollution due to their prevalence in the environment and potential health impacts. The NPAHs considered in this research cover a range of molecular weights and structures, including LMW compounds such as 1-nitronaphthalene, 2-nitronaphthalene, 5-nitroacenaphthene, and 2-nitrofluorene, as well as HMW compounds like 9-nitroanthracene, 9-nitrophenanthrene, 3-nitrophenanthrene, 2-nitrofluoranthene, 3-nitrofluoranthene, 4-nitropyrene, 1-nitropyrene, 2-nitropyrene, 7-nitrobenzo­(a)­anthracene, and 6-nitrochrysene. The full names and corresponding abbreviations of these NPAHs are also provided in Table S3.

The toxic equivalency factor (TEF) approach was employed to assess the toxicity of PAH emissions. Among the 16 target PAHs, benzo­[a]­pyrene (BaP) and dibenzo­[a,h]­anthracene (DBA) have the highest TEFs, both defined as one.^26^ Based on BaP as the reference compound, the toxicities of other PAH congeners were evaluated and expressed as “BaP equivalents (BaPeq)” (Table S3). The TEF values used for each PAH congener were adopted from the widely cited work by Nisbet and LaGoy,^26^ which established a standardized framework for assessing the relative carcinogenic potency of PAHs. However, no standardized toxicity criteria have been established for NPAHs to date. The Office of Environmental Health Hazard Assessment has developed a Potency Equivalency Factor (PEF) procedure to assess the relative potencies of PAHs and PAH derivatives as a group, addressing the impact of carcinogenic PAHs in ambient air, where they are typically present as complex mixtures.^27^ This study adopts the Air Toxics Hot Spots Program Risk Assessment Guidelines and includes the five most toxic NPAHs (5-nitroacenaphthene, 1-nitropyrene, 4-nitropyrene, 6-nitrochrysene, and 2-nitrofluorene) in the overall toxicity assessment (Table S3). The total toxicity of all 16 PAHs and 5 NPAHs in a sample was calculated as the total BaPeq, using the following equation:

1 $$Total\;BaP_{eq}\;\left(\mathrm{ng}/{\mathrm{Nm}}^{3}\right)=\underset{i}{\sum }TEF_{i}\cdot m_{i}$$

where *mi* represents the mass concentration of each PAH or NPAH congener, the mass and BaP_eq_ concentrations of PAHs and NPAHs were calculated under standard conditions (0 °C and 1 atm) and were expressed as *C_PAH_* (ng/Nm^3^) and $C_{BaP_{eq}}$ (ng/Nm^3^), respectively, for further analysis. More analytical details can be found in our previous study.^13^

#### 2.3.2 Sampling and Analysis of PAHs and NPAHs

This study involved three main types of sampling: solid-phase and liquid-phase sampling of pollutants (at Points F, G, H, J, and K) and gas sampling in the flue gas ducts (at Points A, B, C, and D). Pollutants were categorized into dry and wet types. The dry samples included incinerator ash, cooling ash, and fly ash, while the wet samples comprised scrubber sludge and cyclone separator sludge. Sampling commenced when HAWTTS reached stable operating conditions and continued until the end of the designated sampling period. All fly ash generated from each unit was collected separately during this time. Each sampling process was repeated three times to minimize experimental error.

The SCB and CYCD tank suspensions collected 3 × 30 L (approximately 3 × 30 kg) of sludge. Additionally, 3 × 1000 g of ash were collected from beneath the incinerator (Point F), beneath the quenching tower (Point G), and from the baghouse (Point K). The PAHs and NPAHs in the sludge were collected according to the Taiwan EPA standard method (NIEA W790), using glass fiber filters with a pore size of 0.5 μm for particulate matter and glass cartridges packed with polyurethane foam (PUF) for the adsorption of dissolved PAHs and NPAHs. Sampling was conducted at a pump rate of 1 L/min for approximately 30 min. Ultimately, two types of PAH and NPAH samples were collected: particulate PAHs and NPAHs on filter paper and dissolved PAHs and NPAHs adsorbed on PUF.

Flue gas samples were collected via isokinetic sampling according to the United States Environmental Protection Agency (U.S. EPA) Modified Method 23,^28^ using sampling equipment specified in the U.S. EPA Modified Method 5.^29^ Isokinetic sampling adjusts the pressure differential to automatically control the sampling speed, matching it to the flue gas velocity, ensuring representative sampling. The samples were collected continuously under consistent operating conditions, rather than simultaneously. The sampling time for each flue gas sample ranged from 0.5 to 2.5 h. The sampling volume was then standardized to standard conditions (1 atm and 273 K) and represented as 2 to 2.5 N m^3^. Each PAH and NPAH sample included both particulate and gas-phase components. Particulate PAHs and NPAHs were collected on quartz fiber filters, specifically designed to capture particulates in the flue gas. At the same time, gas-phase congeners were trapped using glass cartridges filled with PUF. The quartz fiber filters were ultrasonically cleaned in a dichloromethane bath for 1 h to remove background organic impurities and then dried with nitrogen gas (purity 99.999%). Particulate PAHs in the filter samples were pretreated by Soxhlet extraction for 16 h. The extraction solvent was a mixture of *n*-hexane and dichloromethane (1:1 v/v), which was then concentrated to 200 mL under vacuum, eluted through a silica gel column with *n*-hexane, and finally concentrated to 0.5 mL by nitrogen blow-down. A more detailed description of the sampling and analytical procedures for PAHs and NPAHs, including method detection limits and quality control parameters, is provided in the Supporting Information section titled “Analytical Methods and Quality Control for PAHs and NPAHs.”

#### 2.3.3 Particle Size Distribution (PSD) and Instrumental Analysis

The sampling and analysis methods for particle size distribution (PSD) in this study followed standard procedures established by the U.S. EPA and the California Air Resources Board (CARB Method 501). Coarse particles in the flue gas were first excluded through the cyclone inlet of the sampling probe. Then the PM10 fraction was collected on an eight-stage cascade impactor with different aerodynamic diameter ranges. Eight quartz fiber filters were used, including two 47 mm circular and six annular filters. Notably, quartz liners were employed to ensure a smooth flue gas inlet at high temperatures (429 °C) and avoid corrosion effects at Point A, near the upper temperature limit of USEPA Method 5 (0–449 °C). The hot gas stream sampled was then directed into a heated oven (120 ± 10 °C) containing the eight-stage impactor to prevent PSD modification due to cooling and condensation. The same measurement procedure was applied at other sampling points to limit interstage differences. The PM collected on the filters was used to analyze the PSD, quantify the particulate-phase PAH (FP-PAH) content, and provide backup samples. The mass of PM_10_ was determined using a five-decimal-place microbalance (METTLER TOLEDO XS105DU) in a Class-100 cleanroom. PSD was expressed as the PM mass concentration per standard flue gas sampling volume (Nm^3^). Three replicate samples collected over 180 min (0.81–1.62 N m Nm^3^) were obtained at each sampling point to ensure representative results.

The quantification of PAHs and NPAHs was conducted using gas chromatography–mass spectrometry (GC-MS, Agilent Network 6890 N) with 5975B inert selective detector. For more detailed descriptions, please refer to our previous studies.^13,19^ To evaluate the reliability of the reported removal efficiencies, we conducted an uncertainty analysis considering three primary sources of error: (1) sampling variability, (2) analytical repeatability, and (3) instrument calibration. Field duplicates indicated a relative standard deviation (RSD) of 6–12% for PM mass concentration and 8–15% for PAHs and NPAHs. Based on triplicate injections of calibration standards, analytical precision showed RSDs within ±5%. Instrument calibration uncertainties were incorporated into the propagated error, including flow rate stability (±3%) and weighing precision (±1%). By combining these sources using standard error propagation methods, the overall uncertainty in removal efficiency calculations was estimated to range between ±7.5% and ±13.2%, depending on the sampling point and pollutant species. These values have been used to assess the robustness of our conclusions and are considered within acceptable bounds for field-scale combustion studies.

### 2.4 Evaluation of PAH and NPAH Removal Performance in the HAWTTS

Evaluation of PAH and NPAH Removal Performance in the HAWTTS (η) was calculated. This study considers the closed-loop nature of the system, where toxic residues are reintegrated into the waste feedstock. The inputs to the incinerator include waste (Point E) and SFA from the SCB (Point H), CYCD (Point J), and BH (Point K). The outputs from the incinerator are the flue gas at Point A, bottom ash at Point F, and quenching sludge at Point G. The input to the APCDs is the flue gas at Point A, and the output is the flue gas at Point D. The input for the overall HAWTTS is the same as that of the incinerator, with its output corresponding to that of the APCDs. The removal efficiencies of the incinerator (*η_inc_*) , APCDs $\left(\eta_{APCDs}\right)$ , and HAWTTS (*η*_*HAW*TTS_) were calculated as follows:

2 $$\eta_{inc,i}=\frac{\left(f_{E,i}+f_{H,i}+f_{J,i}+f_{K,i}\right)-\left(f_{A,i}+f_{F,i}+f_{G,i}\right)}{f_{E,i}+f_{H,i}+f_{J,i}+f_{K,i}}\times 100\%$$

3 $$\eta_{APCDs,i}=\frac{f_{A,i}-f_{D,i}}{f_{A,i}}\times 100\%$$

4 $$\eta_{HAWTTS,i}=\frac{\left(f_{E,i}+f_{H,i}+f_{J,i}+f_{K,i}\right)-\left(f_{D,i}+f_{F,i}+f_{G,i}\right)}{f_{E,i}+f_{H,i}+f_{J,i}+f_{K,i}}\times 100\%$$

where *i* denotes different congeners of PAHs or NPAHs, and *f* represents the mass flow of PAHs at the corresponding sampling points.

The removal efficiencies of individual APCD units were calculated based on the mass concentrations of pollutants in the flue gas. The removal efficiencies for the SCB $\left(\eta_{SCBs}\right)$ , CYCD (*η_CYCD_*), and BH (*η_B__H_*) are given by

5 $$\eta_{SCBs}=\frac{C_{A}-C_{B}}{C_{A}}\times 100\%$$

6 $$\eta_{CYCD}=\frac{C_{B}-C_{C}}{C_{B}}\times 100\%$$

7 $$\eta_{BH}=\frac{C_{C}-C_{D}}{C_{C}}\times 100\%$$

where *C*_*A*_, *C_B_*, *C_C_*, and *C_D_* represent the mass concentrations of PAHs and NPAHs in the flue gas at Points A, B, C, and D, respectively.

To further assess the actual performance of the HAWTTS in pollutant management, net discharge (*d_net_*) was calculated as follows:

8 $$d_{net}=\left(f_{D,i}+f_{F,i}+f_{G,i}\right)-\left(f_{E,i}+f_{H,i}+f_{J,i}+f_{K,i}\right)$$

where (*f*_*E*,*i*_ + *f*_*H,i*_ + *f_J,i_* + *f*_*K,i*_) represents the total input of the PAH or NPAH congener into the HAWTTS, and (*f_D,i_* + *f_F,i_* + *f_G,i_*) represents the total output. A negative value of *d_net_* indicates a net reduction in emissions, while a positive value indicates a net increase.

## 3 Results and Discussion

### 3.1 Removal Efficiency of PAH and NPAH in the HAWTTS

The overall removal efficiencies of total PAHs achieved by the incinerator, APCDs, and the complete HAWTTS were 98.7%, 63.9%, and 99.2%, respectively (see Figure 2). This highlights the critical role of the incinerator in the thermal decomposition of PAHs. PAH congener removal in the incinerator ranged from 95.8% to 99.9%. However, the APCDs exhibited lower removal efficiencies, particularly for LMW PAHs such as NaP, AcPy, AcP, and Flu, and for the most toxic congeners, IND and DBA. DBA notably showed no removal within the APCDs.

**Figure 2:**
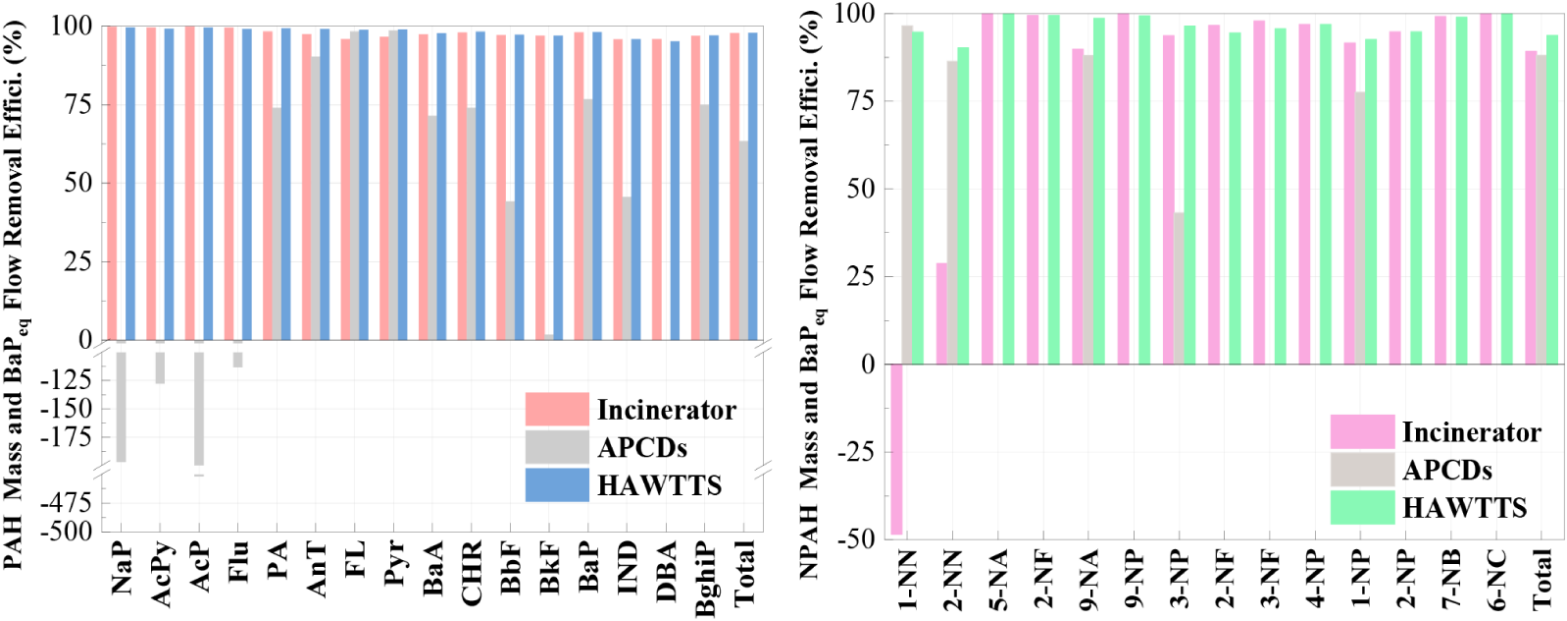
PAH and NPAH congener removal efficiency for incinerator, APCDs, and HAWTTS.

The lower removal efficiency of LMW PAHs in the APCDs suggests their potential formation or release within these units. This can be attributed to several of the following factors: (1) Volatility and desorption: LMW PAHs, due to their higher vapor pressures and lower boiling points, tend to remain in the gas phase and may desorb from particle surfaces under the elevated and fluctuating temperatures within APCDs, particularly in the baghouse.^30,31^ (2) Particle capture limitations: Since APCDs primarily target particulate-bound pollutants, gaseous-phase LMW PAHs are inherently less effectively captured. (3) Secondary formation: Conditions within the APCDs, such as varying temperatures, residence times, and the presence of reactive species, may facilitate the secondary formation of PAHs from incomplete combustion byproducts or through reactions involving residual organic compounds adsorbed on particulates.^32^ This phenomenon suggests that, rather than effectively capturing LMW PAHs, the APCDs may inadvertently contribute to their formation or release, reducing the overall control efficiency for these specific compounds.

For NPAHs, the removal efficiencies were 89.2%, 88.1%, and 93.7% for the incinerator, APCDs, and HAWTTS, respectively (see Figure 2), with lower removal rates than PAHs. Although the incinerator and APCDs demonstrated similar total NPAH removal performances, the dominant mechanisms differ between these units. NPAHs were primarily removed in the incinerator through high-temperature thermal decomposition and radical-driven oxidation processes, particularly via hydrogen abstraction and ring-opening reactions. In contrast, the APCDs achieved NPAH removal mainly through the physical capture of particle-bound species by filtration and inertial impaction, with minimal chemical transformation expected at the lower temperatures present in these devices. High removal rates were observed for several NPAH congeners, such as 5-NA, 9-NP, and 6-NC, all reaching 100%. However, 1-NN showed a negative removal rate, indicating its formation within the incinerator. This is likely due to the thermal decomposition or partial oxidation of higher-ring NPAHs, leading to the formation of lower-ring compounds like 1-NN during incineration.^33^

Importantly, HAWTTS achieved a net removal of PAHs, NPAHs, and BaP_eq_, with net removal rates of −1.03 × 10^12^ ng/h, 2.56 × 10^9^ ng/h, and −1.93 × 10^10^ ng BaP_eq_/h for BaP_eq_, respectively. This demonstrates the closed-loop system’s effectiveness in reducing these pollutants’ overall emissions, even considering the reintegration of APCD residues.

### 3.2 Formation and Decomposition of PAHs and NPAHs in the Incinerator

#### 3.2.1 Effect of SFACC on PM Size Distribution

A clear comparison of the PM size distribution in the flue gas downstream of the incinerator (Point A) with and without SFACC operation reveals the substantial impact of residue reintegration. Without SFACC, the total PM emissions at Point A were relatively low, measured at 34.9 mg/Nm^3^. However, after sludge and fly ash reintegration through SFACC, the total PM emissions dramatically increased to 84,300 mg/Nm^3^ (see Figure 3), representing an increase by several orders of magnitude. This substantial increase indicates that most of the PM in the flue gas originates from the reintroduced SFA. The significant increase in PM emissions is attributed to the volatilization of inorganic components and residual metals from the recycled SFA. During combustion, compounds such as metal chlorides, sulfates, and other ash-forming minerals are released into the gas phase and nucleate as the flue gas cools. These primary particles grow further by condensation of semivolatile species and coagulation, significantly increasing the total particulate mass observed at Point A.^34^ Moreover, metallic species in the SFA may act as catalysts in the formation and pyrolysis of PAHs and NPAHs during incineration.

**Figure 3:**
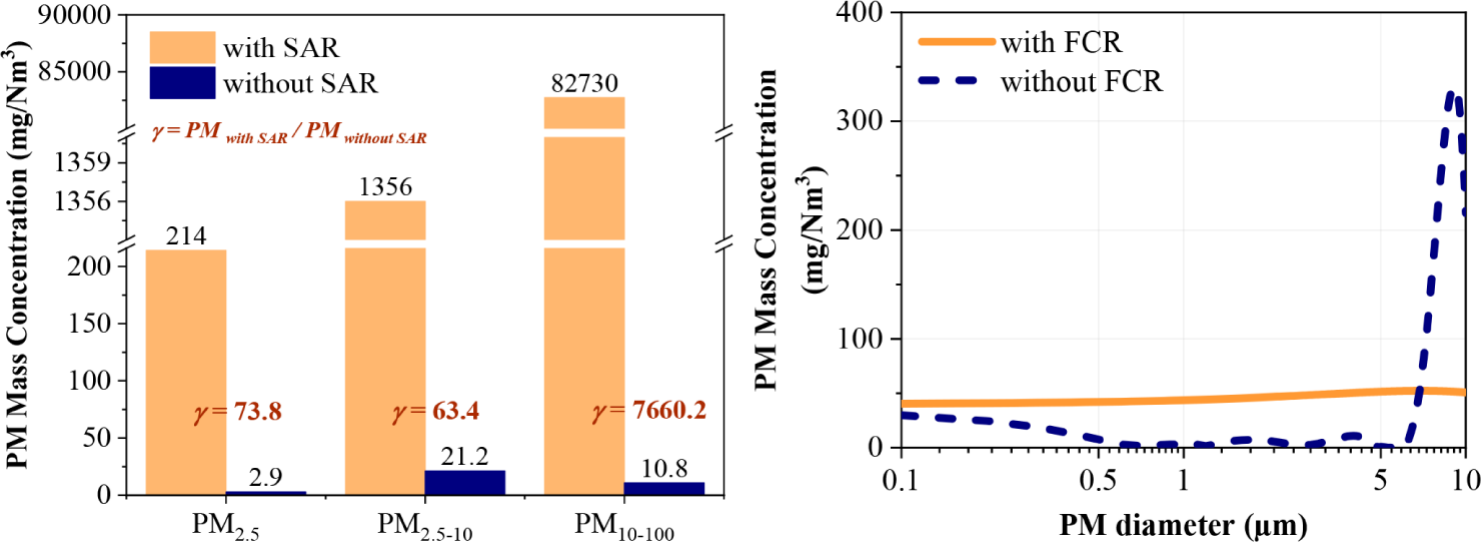
PM size distribution in the combustion exhaust with/without SFACC coincineration.

With SFACC operation, the mass concentrations of PM_2.5_, PM_2.5–10_, and PM_10–100_ were 73.8, 63.4, and 7660.2 times higher, respectively, than those without SFACC (see Figure 3). This indicates that SFACC significantly increased the mass concentration of PM in both the fine and coarse particles. However, in the *d*_*p*_ = 6.9–10 μm range, conventional operation without SFACC exhibited higher PM mass concentrations. The elevated concentration of fly ash reintroduced into the incinerator increases the number of available nucleation sites for particle growth. As the flue gas cools downstream, the supersaturation of semivolatile organic leads to their preferential condensation onto existing particle surfaces. Surface-active materials in the recycled ash, such as metallic oxides, minerals, and carbonaceous residues, lower the energy barrier for heterogeneous nucleation and facilitate multilayer adsorption. These combined thermodynamic and surface chemical processes promote enhanced particle growth through condensation, adsorption, and coagulation, significantly increasing the PM_**10**–**100**_ mass concentration.^35^ The impact of this increased particle concentration on PM removal by the APCDs will be discussed in subsequent sections.

#### 3.2.2 Flow Rate and Distribution of PAH and NPAH Congeners

Analyzing the distribution of PAHs and NPAHs across various locations within the HAWTTS reveals distinct patterns in mass flow rates and congener compositions. In addition, a detailed breakdown of individual PAH congeners and the relative proportions of LMW and HMW PAHs in the waste and recycled ash streams is provided in Table S8. The total PAH mass flow input to the incinerator was 1.04 × 10^10^ ng/h, with waste and SFA contributing 44.0% and 56.0%, respectively (Figure 4).

**Figure 4:**
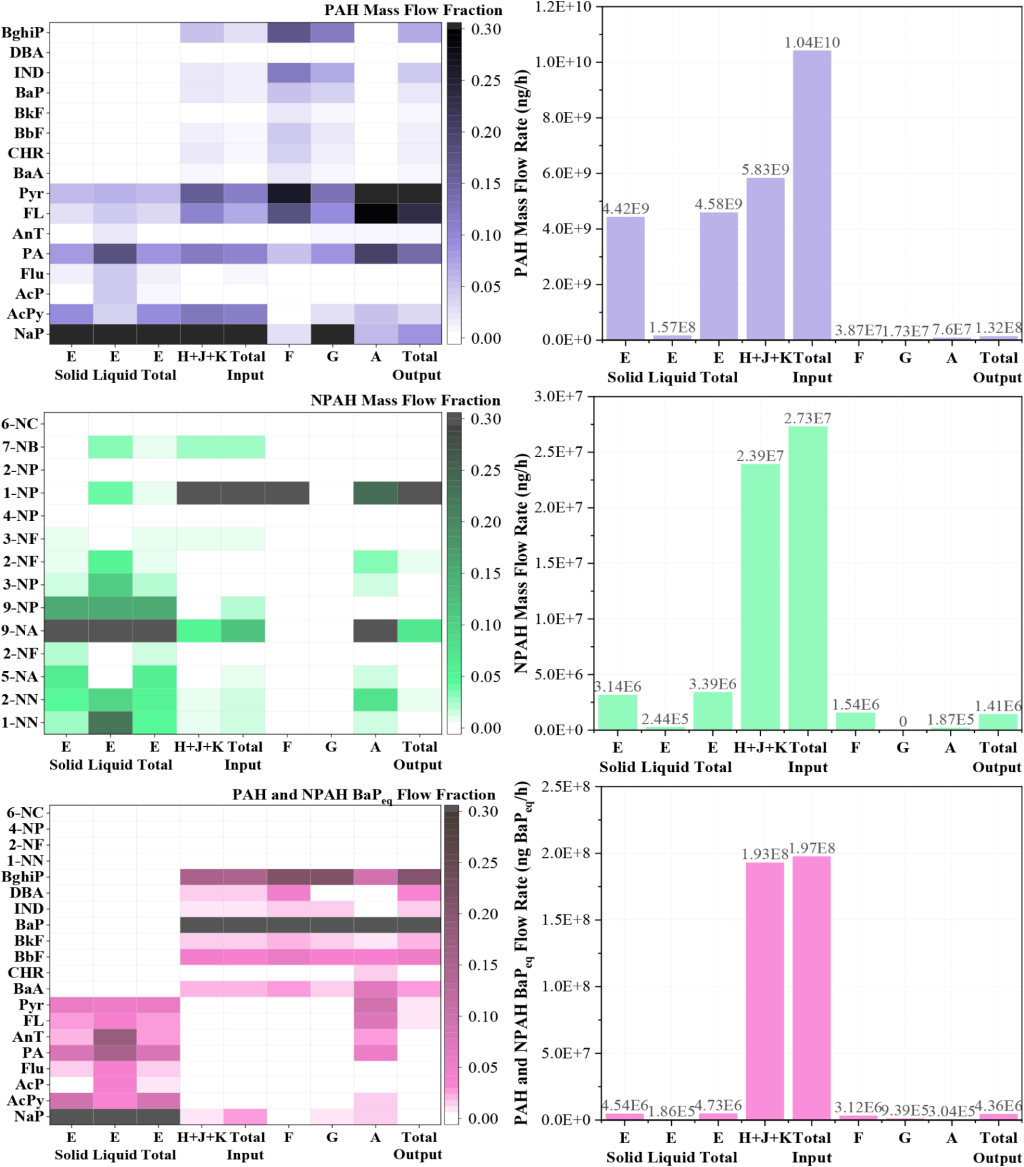
PAH and NPAH input/output mass flow, congeners, and BaP_eq_ of the incinerator.

For NPAHs, the contributions from waste and SFA were 12.4% and 87.6%, respectively. Regarding toxicity, the contributions of BaPeq from waste and SFA to the total BaPeq were 2.4% and 97.6%, respectively. This indicates that the secondary pollution generated by the APCDs has significantly higher PAH and NPAH toxicity than raw waste. This is likely due to the accumulation of more toxic, higher molecular weight PAHs and NPAHs in the APCD residues. Additionally, PAHs and NPAHs in the raw waste are primarily concentrated in the solid phase, largely due to their low water solubility.^36^

The congener distribution heatmap (Figure 4) provides further insights. Darker shading represents higher contributions of a specific PAH or NPAH congener. In the raw waste, LMW PAHs such as NaP, AcPy, PA, and Pyr, along with NPAHs like 9-NA, 9-NP, 5-NA, 2-NN, and 1-NN, dominate the profiles. In contrast, the SFA (H + K + J) exhibits a different pattern, with a significant increase in the proportion of HMW PAHs and NPAHs, particularly PAH congeners such as FA and Pyr, and NPAH congeners such as 1-NP, 9-NA, and 7-NB. The proportion of highly toxic substances like BghiP, IND, and BaP also increases in the SFA, making it the major contributor to the PAH toxicity in the incinerator feedback.

Several factors contribute to this shift in congener distribution. (1) Thermal decomposition behavior: At combustion temperatures above ∼900 °C, PAHs undergo thermal degradation via hydrogen abstraction, ring-opening, and fragmentation reactions. However, HMW PAHs exhibit greater thermal stability than LMW PAHs, resulting in their preferential persistence in the residual ash after incineration. Furthermore, the adsorption and condensation of PAHs onto ash surfaces are influenced by the ash’s surface area, porosity, and chemical composition, with carbonaceous and porous structures providing favorable sites for the selective retention of HMW PAHs due to their larger molecular size and lower volatility. (2) Radical-driven oxidation: The presence of reactive radicals, particularly hydroxyl (OH•) and atomic oxygen (O•), promotes the oxidation and breakdown of PAHs during high-temperature combustion. Under uniform and moderate-temperature MILD combustion conditions, these radicals facilitate gradual oxidation, minimizing the formation of new PAH and NPAH species.^6,37^ (3) Adsorption: Residual carbonaceous materials in the ash, characterized by high surface area and porous structures, provide effective substrates for the adsorption and condensation of PAHs and NPAHs. The ash’s hydrophobicity, porosity, and mineral composition influence both the adsorption capacity and selectivity, with HMW PAHs exhibiting stronger adsorption due to their lower volatility, larger molecular size, and higher affinity for carbon-rich particulate surfaces.^38,39^ (4) Oxidation resistance: Highly toxic PAH congeners like BghiP, IND, and BaP are more resistant to oxidation and less volatile due to their higher molecular weight, making them more prone to partitioning into the solid phase.^40^

Observing the distribution of PAH and NPAH congeners in the bottom ash (F) and quenching ash (G) reveals a further increase in the mass fractions of higher-ring PAHs and NPAHs, such as FA, Pyr, BghiP, IND, BaP, and 1-NP (see Figure 4). This suggests that high-temperature thermal treatment leads to the formation of PAHs and NPAHs with higher toxicity.^32^ The metal substances in the fly ash may also catalyze the formation of these high-ring, high-toxicity compounds.^41^

At Point A, where the flue gas remains at relatively high temperatures, both low-ring and high-ring PAH and NPAH congeners are detected. Unlike downstream sampling points, Point A contains a significant gaseous-phase fraction. Due to their higher saturation vapor pressures and lower molecular weights, low-ring PAHs and NPAHs preferentially volatilize into the gas phase, resulting in their relatively higher concentrations at this location. Meanwhile, as the flue gas begins to cool downstream of the combustion zone, the formation of high molecular weight PAHs and NPAHs is favored through hydrogen abstraction and acetylene addition mechanisms, radical recombination, and surface-catalyzed reactions on fly ash particles.^42^ These processes contribute to the progressive enrichment of HMW species observed at subsequent lower-temperature sampling points.

While the incineration process significantly reduces the total mass of PAHs and NPAHs, the congener distribution shifts from being dominated by low-toxicity to high-toxicity compounds. This phenomenon is primarily attributed to the preferential adsorption of less volatile, highly toxic congeners onto particulate matter, particularly in larger particle fractions enhanced by fly ash reintegration. Such shifts impact the overall emission characteristics and elevate potential health risks.

### 3.3 Distribution of PAH and NPAH Congeners and Particle Size in the APCDs

#### 3.3.1 PAH and NPAH Congener Distribution in Flue Gas

##### 3.3.1.1 Scrubbers (SCB)

After the flue gas passes through the SCB, the total PAH mass concentration decreases by 30.1%. However, this decrease is not uniform across phases. The concentration of particulate PAHs is reduced by 70.4%, while the concentration of gaseous-phase PAHs increases by 113%. This shift results in a change from 78% of the total PAH mass being associated with the particulate phase at Point A (incinerator outlet) to 67.0% being in the gaseous phase at Point B (SCB outlet) (Figure 5). This suggests that either volatilization of particulate PAHs into the gas phase occurred within the SCB, or new gas-phase PAHs were generated during the temperature drop from 950 to 400 °C. Moreover, the temperature reduction in the SCB induces competing volatilization and condensation processes for semivolatile organic compounds. As particle-bound PAHs experience decreased surface binding strength at elevated temperatures, some may volatilize into the gas phase, especially low molecular weight congeners. Concurrently, cooling flue gas promotes the supersaturation and subsequent condensation of semivolatile species onto particulate surfaces, enhancing their removal by downstream air pollution control devices. This dynamic balance underscores the critical role of temperature control in regulating organic pollutants’ phase distribution, transformation, and ultimate removal efficiency.

**Figure 5:**
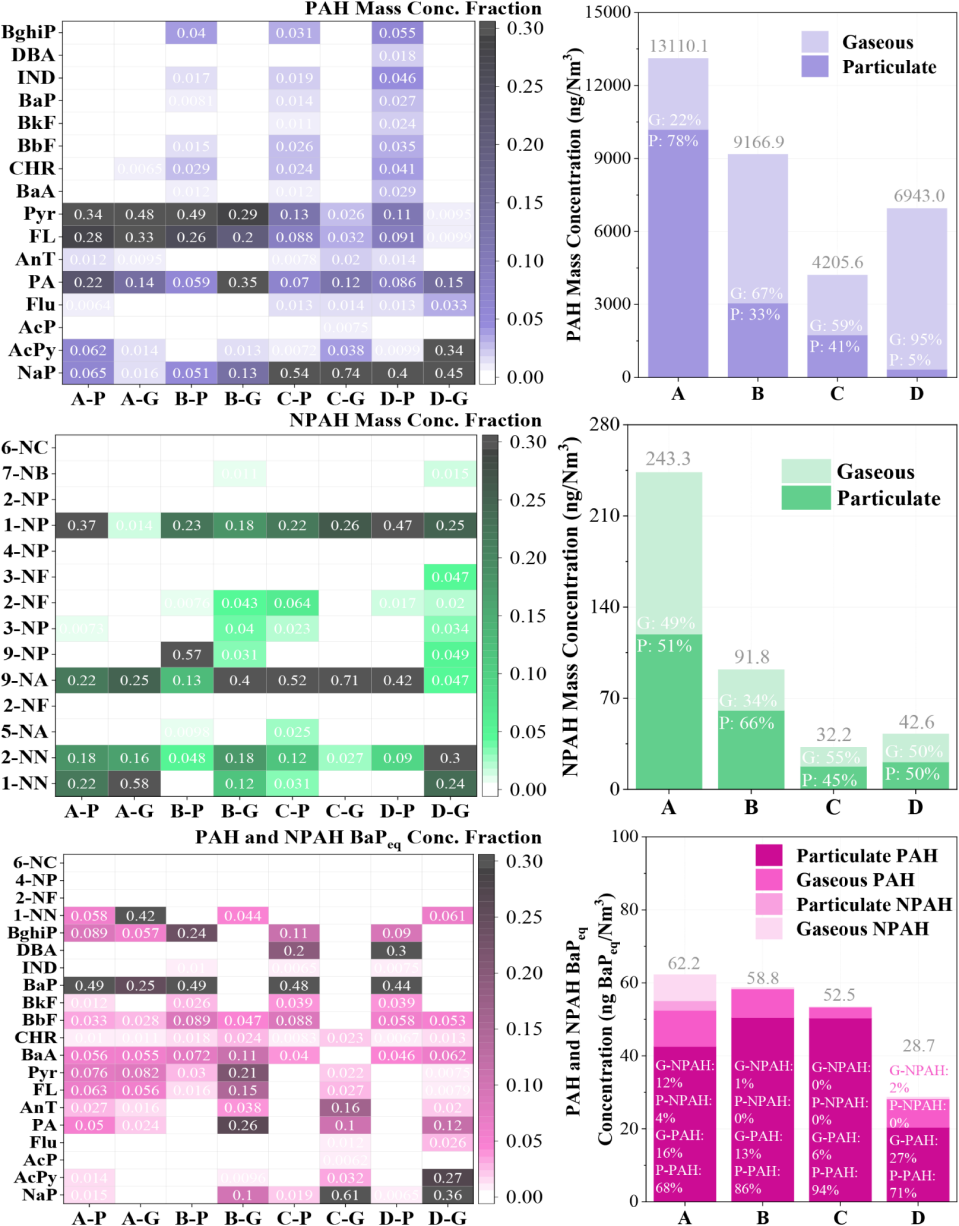
PAH and NPAH congener distribution in the flue gas of APCDs.

The congener distribution of PAHs shows that the fingerprints of particulate and gaseous PAHs at Point A are similar, dominated by Pyr, FL, and PA. At this high-temperature stage, low-ring PAHs are more prevalent in the particulate phase than in the gaseous phase, as their volatility is less prominently expressed. The congener distribution at Point B is generally similar to that at Point A, where Pyr, FL, and PA also dominate. However, at Point B, the mass fraction of higher-ring PAHs in the particulate phase increases significantly, notably BghiP, IND, and BaP. This suggests that the SCB primarily removes low-ring PAHs in the particulate phase. In contrast, the newly generated gaseous-phase PAHs are mainly composed of low-ring PAHs due to phase transition, potentially influenced by the increased fly ash concentration in the flue gas.

In contrast to PAHs, both particulate and gaseous NPAH mass concentrations decrease after the SCB, with the gaseous fraction showing a more significant reduction of 73.8% (see Figure 5). The proportion of gaseous NPAHs decreases from 49% at Point A to 34% at Point B. The particulate and gaseous phases at Point A have similar NPAH congener distributions. However, the particulate NPAHs dominate the high-ring 1-NP (37% by mass). At the same time, in the gaseous phase, 1-NN accounts for 58%, aligning with the expectation that more volatile, lower-ring NPAHs are primarily found in the gaseous phase.

After scrubbing, Point B’s overall NPAH congener distribution remains similar, still dominated by 1-NP, 9-NP, 9-NA, and 2-NN. However, the proportion of 9-NN in the particulate phase increases sharply, suggesting its possible formation within the SCB (see Figure 5). The reduction in toxicity from Point A to Point B is insignificant, decreasing by only 5.5%. Notably, the concentration of particulate PAH BaPeq increases significantly, from contributing 68.0% of the total BaPeq at Point A to 86.0% at Point B. The toxicity is mainly contributed by PAHs, with NPAHs contributing 16.0% of the toxicity at Point A and only 1% at Point B. The PAH and NPAH congener toxicity distribution shows that at Point A, the toxicity in the particulate phase is mainly contributed by BaP. In contrast, in the gaseous phase, it is primarily contributed by 1-NN and BaP. After passing through the SCB, BaP exhibited relatively low removal efficiency, which may be attributed to its high molecular weight, thermal stability, and low volatility. In contrast, 1-NN is effectively removed. This highlights 1-NN as a concern in hazardous waste incineration and emphasizes the need for improved SCB removal efficiency for high-ring PAHs.

##### 3.3.1.2 Cyclone Demister (CYCD)

From Point B to Point C, the total PAH mass concentration decreases by 54.12%, with particulate and gaseous NPAH mass concentrations decreasing by 43.0% and 59.6%, respectively (Figure 5). The congener distribution of PAHs shows that the distributions in both the particulate and gaseous phases at Point B are similar to those at Point C, with almost no high-ring PAH congeners in the gaseous phase. A significant reduction in fluoranthene and pyrene concentrations was observed after the gas stream passed through the cyclone-based device. This resulted in a shift in dominant PAH congeners from midring to low-ring species. This may be associated with physical removal processes influenced by particle size, morphology, or surface properties.^43^ The CYCD more effectively removes substances attached to larger-diameter PM or water droplets. However, the CYCD has a poor removal rate for some high-ring particulate PAH congeners, such as BaP, BkF, and BbF.

The CYCD achieves an overall NPAH removal rate of 64.9%, with a higher removal rate for particulate NPAH (76.1%) than gaseous NPAH (43.3%). The CYCD mainly removes 9-NP from the particulate NPAHs but has a poor removal rate for 9-NA. It removes gaseous NPAHs more uniformly, with 9-NA remaining the dominant congener. The concentration and size of PM in the flue gas likely influenced the removal rate. Although the CYCD achieves a satisfactory removal rate for overall PAH and NPAH mass concentrations, the BaP_eq_ removal rate is only 11.0%. This is attributed to the high contribution of solid-phase PAHs to BaP_eq_ and the poor removal rate for highly toxic congeners.

##### 3.3.1.3 Baghouse (BH)

After the flue gas passes through the BH (from Point C to Point D), the total PAH and NPAH mass concentrations increase by 65.1% and 32.3%, respectively, while the total BaPeq decreases by 45.3%. This increase is likely associated with the so-called “memory effect” of the baghouse, whereby previously adsorbed semivolatile compounds may be rereleased under changing conditions. Fluctuations in temperature, pressure, or gas flow may lead to desorption from the filter media or disturbance of the filter cake, particularly affecting low molecular weight congeners with higher volatility and weaker adsorption affinity.^44^ However, this rerelease favors lower toxicity compounds, resulting in a decrease in total BaP_eq_.

The BH shows a significant removal effect for particulate PAHs, but for the remaining particulate PAHs in the flue gas, the mass fractions of high-ring, highly toxic PAH congeners such as BghiP, DBA, IND, BaP, BkF, BbF, CHR, and BaA increase. Conversely, the gaseous PAH mass concentration increases significantly, primarily dominated by low-ring AcPy and NaP. This indicates that the PAHs rereleased from the BH are mainly in the gaseous phase and suggests that the PAH congeners most likely to be rereleased during the memory effect are those with low-ring and low-toxicity properties.

From Point C to Point D, the dominant particulate NPAH congeners remain 9-NA and 1-NP, however, after passing through the BH, gaseous NPAHs increase, including 9-NP, 2-NP, 3-NF, and 7-NB. Overall, although the BH increases the mass concentrations of PAHs and NPAHs due to the memory effect, it effectively reduces the overall toxicity in the flue gas.

#### 3.3.2 Particle Size Distribution of PM in the APCDs

The particle size distribution analysis was limited to PM_10_ due to the relevance of 10 μm particles to human health risk. Notably, submicron and fine particles (PM_1_ and PM_2.5_) contributed disproportionately to total PM mass, and the observed removal efficiencies of each APCD unit corresponded well with their effectiveness in targeting specific size fractions, thereby influencing overall mass reduction.

##### 3.3.2.1 Scrubber (SCB)

The reintegration of APCD residues significantly increased the particle size and mass concentration of PM at Point A (incinerator outlet). However, after scrubbing with the SCB, PM_10–100_ was removed entirely, and the total PM mass concentration was reduced to 63.9 mg/Nm^3^. This demonstrates the excellent efficiency of the SCB in removing larger particles, effectively mitigating the increase in particle size and mass concentration caused by SFACC.

The PSD analysis reveals that SFACC leads to merging the nucleation and accumulation mode peaks. In the size range of *d*_*p*_ = 0.1–1.9 μm, a relatively high PM mass concentration is observed, peaking at 14.7 mg/Nm^3^ (see Figure 6). The coarse particle peak reaches a maximum concentration of 19.8 mg/Nm^3^ at *d*_*p*_ = 6.3 μm. In contrast, without SFACC, the peak PM mass concentrations are smaller, with the nucleation peak at 3.2 mg/Nm^3^ at *d*_*p*_ = 1.1 μm, and the accumulation and coarse particle peaks at 6.3 mg/Nm^3^ and 1.4 mg/Nm^3^ at *d*_*p*_ = 3.2 μm and *d*_*p*_ = 8.6 μm, respectively. This is because, under SFACC, the PM_10_ mass concentration in the flue gas is inherently higher than that under no SFACC. Additionally, the high PM mass concentration in the flue gas during scrubbing provides more nuclei, facilitating the adsorption and condensation of VOCs, potentially further increasing particle size.

**Figure 6:**
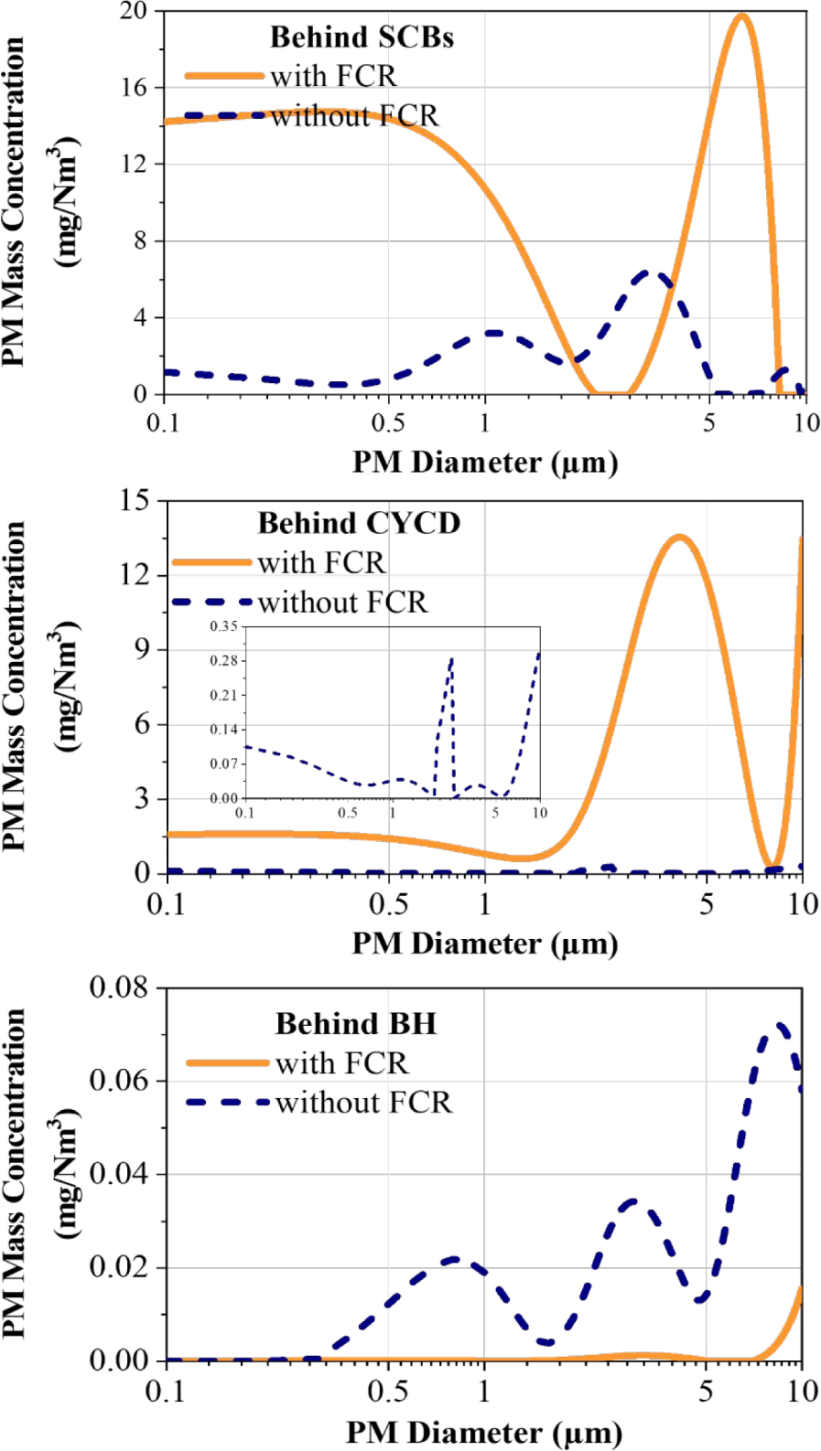
Particle size distribution of PM across the APCDs.

##### 3.3.2.2 Cyclone Demister (CYCD)

After passing through the CYCD, the overall PM mass concentration decreases under SFACC operation, especially in the range of *d*_*p*_ = 0.1–1.4 μm, where the peak value is reduced by 89% (Figure 6). However, particles with *d*_*p*_ > 10 μm are formed within the CYCD. This is attributed to the turbulent conditions within the CYCD, which promote collisions and adhesion between particles and water droplets, facilitating the formation of larger particles.^45^ A similar phenomenon is observed without SFACC, where the CYCD significantly reduces PM mass concentration but also promotes the formation of larger particles (*d*_*p*_ > 10 μm). Facilitating the growth of fine particles is beneficial for reducing the emission of respirable particulate matter.

##### 3.3.2.3 Baghouse (BH)

After passing through the BH, the particle size within the PM10 range is significantly reduced under both SFACC and non-SFACC conditions. The PM mass concentration is notably reduced to a lower level under SFACC conditions. The peak values of the nucleation and accumulation modes are only 7 × 10^–5^ mg/Nm^3^ and 0.001 mg/Nm^3^, respectively. In contrast, without SFACC, the peak PM mass concentrations are 0.02 mg/Nm^3^ (nucleation mode, *d*_*p*_ = 0.8 μm), 0.03 mg/Nm^3^ (accumulation mode, *d*_*p*_ = 2.9 μm), and 0.07 mg/Nm^3^ (coarse particle mode, *d*_*p*_ = 8.4 μm).

The combination of the BH with activated carbon injection achieves satisfactory particulate filtration. The higher input PM concentration under SFACC conditions further enhances the removal efficiency of the BH. This is likely due to the dynamic behavior of particulate filtration within the BH. At elevated particulate concentrations, deposited particles gradually form a cohesive filter cake on the surface of the filter media, which acts as a secondary filtration layer. The buildup of this layer enhances particle interception by reducing pore size, increasing surface area, and promoting mechanical sieving and diffusion-based capture.^46^ A thicker filter cake can also lead to improved removal of fine and coarse particles due to its increased depth and surface area, providing more sites for particle interception, impaction, and diffusion.

### 3.4 Distribution of PAH and NPAH Congeners in Sludge and Ash from APCDs

Analyzing the PAHs and NPAHs in the sludge and ash generated by the APCDs provides valuable insights into the potential hazards of secondary pollution. It enhances our understanding of the removal mechanisms of these pollutants. The PAH mass flow in the ash from the BH (Point K) is the highest, reaching 3.2 × 10^9^ ng/h, while the sludge from the CYCD (Point J) has the lowest PAH mass flow at 4.7 × 10^8^ ng/h (see Figure 7).

**Figure 7:**
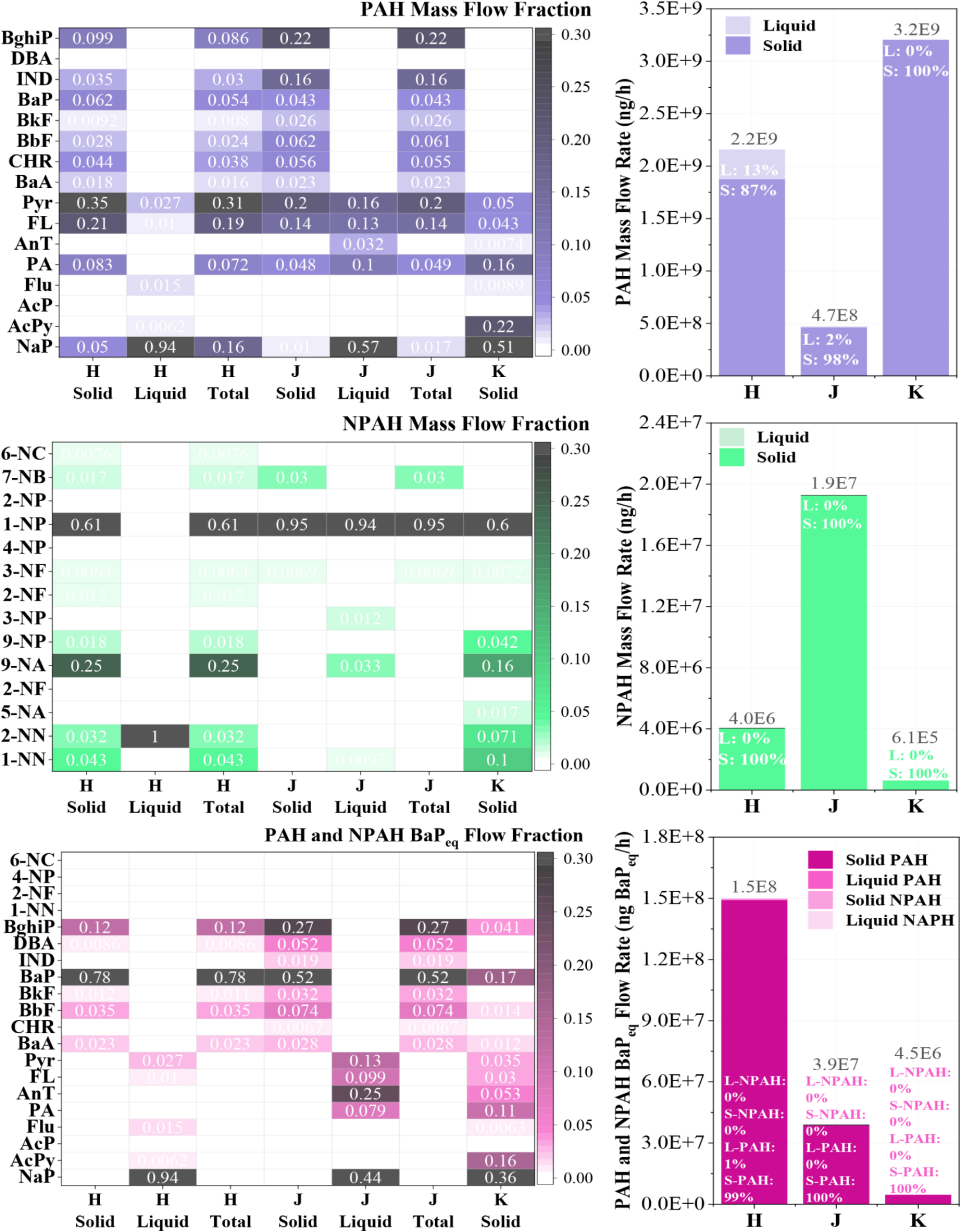
PAH and NPAH congener flow rate in the SFA from APCDs.

The SFA at Points H (SCB) and J (CYCD) exists in a solid–liquid mixed state, whereas the ash at Point K (BH) is in a solid state. Due to the low solubility of PAHs in water, PAHs are primarily concentrated in the solid phase, with only 13% and 2% of PAHs present in the liquid phase at Points H and J, respectively.^47^ A comparison of PAH congeners between the solid and liquid phases reveals that the liquid phase is predominantly composed of NaP. In contrast, the solid phase is mainly composed of Pyr and FL. This pattern is similar to the dominant PAH congeners found in the flue gas at Point A, indicating that this portion of PAHs is directly removed from the flue gas.

The solid phase at Points H and J is dominated by high-ring PAH congeners, indicating that the SFA from the SCB and CYCD is highly toxic. In contrast, the fly ash discharged from the BH (Point K) primarily comprises low-ring PAH congeners. Since NPAHs are also poorly soluble in water,^48^ all NPAHs in the SFA are concentrated in the solid phase. Among the SFA samples, the sludge from the CYCD (Point J) has the highest NPAH content, with 95% being 1-NP. Similarly, the sludge and ash from the SCB (Point H) and BH (Point K) are also primarily composed of 1-NP. This aligns with the NPAH congener distribution observed at Point A, indicating that the NPAHs in the SFA mainly originate from those removed from the flue gas.

The BaP_eq_ flow fraction of PAH and NPAH congeners suggests that a significant portion of PAH toxicity is concentrated in the solid phase of the residues, particularly within the scrubber sludge (Point H). Although the fly ash from the BH (Point K) has the highest contribution to the mass flow of PAHs in the SFA, its contribution to overall toxicity is the lowest due to its low-ring PAH composition. Furthermore, the emission levels of NPAHs in the SFA are much lower than those of PAHs, and their contribution to toxicity is nearly negligible compared to that of PAHs.

## 4 Conclusions

This study investigated an innovative full-scale hazardous waste thermal treatment system (HAWTTS) employing GASMILD combustion to enhance waste treatment efficiency and reduce emissions. The research focused on the effects of blending APCD-generated residues on PM, PAHs, and NPAHs emissions. Comprehensive sampling and analysis were conducted at key locations within the HAWTTS to assess changes in particle size distribution and toxicity. The main findings are summarized as follows:

### 4.1 High Removal Efficiency

The incinerator, APCDs, and the overall HAWTTS demonstrated high removal efficiencies for PAHs and NPAHs. The incinerator played a key role in PAH decomposition, achieving a 98.7% removal efficiency for total PAHs. The overall HAWTTS effectively managed secondary pollutants generated by the APCDs, achieving a net removal of 1.03 × 10^12^ ng/h of PAHs, 2.56 × 10^9^ ng/h of NPAHs, and 1.93 × 10^10^ ng BaP_eq_/h of total BaP_eq_.

### 4.2 Impact of SFACC on PM

It significantly increased the mass concentrations of PM_2.5_, PM_2.5–10_, and PM_10–100_ by 73.8, 63.4, and 7,660 times, respectively, compared to operation without SFACC, highlighting the significant impact of residue reintegration on particle size distribution.

### 4.3 Shift Toward Higher Toxicity

High-temperature thermal treatment led to PAHs and NPAHs, predominantly composed of higher molecular weight, more toxic congeners. The fingerprint distribution of PAHs and NPAHs in the SFA showed a shift toward these higher molecular weight compounds in the incinerator bottom ash, with a greater proportion of highly toxic substances such as BghiP, IND, and BaP. This makes the reintroduced SFA a major contributor to PAH toxicity in the system.

### 4.4 APCD Performance

The SCB demonstrated high efficiency in removing larger particles (PM_10–100_) and low-ring particulate-phase PAHs but showed limited effectiveness in removing newly generated gaseous-phase PAHs and high-ring PAHs.

### 4.5 CYCD Limitations

The CYCD exhibited a poor removal rate for high-ring particulate PAHs such as BaP, BkF, and BbF. Its overall NPAH removal rate was 64.9%, with a higher efficiency for particulate NPAHs (76.1%) than gaseous NPAHs (43.3%).

### 4.6 BH Memory Effect

After the flue gas passed through the BH, total PAH and NPAH mass concentrations increased by 65.1% and 32.3%, respectively, due to the memory effect. However, the total BaPeq decreased by 45.3%, suggesting selective desorption and phase changes within the BH, with the rerelease of predominantly lower-toxicity compounds.

This study demonstrates that integrating SFACC under GASMILD conditions within the HAWTTS system significantly reduces net emissions of PAHs, NPAHs, and BaP_eq_, promoting closed-loop pollutant control and offering a scalable, sustainable strategy for improving hazardous waste treatment and informing future environmental management practices.

## Supplementary Material



## Notes

(1) The properties of wastes, and the HAWTT system; (2) basic information and operating condition of HAWTTS units; (3) the primary information (abbreviation, toxicity equivalent factors) of PAH and NPAH homologues; (4) sampling locations and conditions within the HAWTTS facility; (5) computational fluid dynamics simulation analysis of operational conditions; (6) analytical methods and quality control for PAHs and NPAHs; (7) method detection limits and quality control parameters for PAHs; (8) method detection limits and quality control parameters for NPAHs; (9) mass flow rates and relative contributions of individual PAH congeners, LMW PAHs, and HMW PAHs in the SFA streams (PDF)

## Abbreviations

**Glossary**: **Full name**
APCDs: air pollution control devices
BH: baghouses
CYCD: cyclonic demisters
GASMILD: gasification-moderate or intense low-oxygen dilution
HAWTTS: hazardous waste thermal treatment systems
HMW: higher molecular weight
LMW: lower molecular weight
MILD: moderate or intense low-oxygen dilution
NPAHs: nitro-polycyclic aromatic hydrocarbons
PAHs: polycyclic aromatic hydrocarbons
PM: particulate matter
PSD: particle size distribution
SCBs: scrubbers
SFA: sludge and/or fly ash
SFACC: sludge and fly ash circular combustion
SVOCs: semivolatile organic compounds
U.S. EPA: United States Environmental Protection Agency
