# Supplementary material for: Significant Reduction of Systematic PAH and NPAH Discharges from Hazardous Waste Treatment by the Advanced Scrubbing and Circular GASMILD Combustion
Source: ACS Omega. 2025 Sep 26;10(39):44922–36. doi: 10.1021/acsomega.5c01462 (PMC12508921; doi:10.1021/acsomega.5c01462)
Supplement: Supplementary file 1 [file ao5c01462_si_001.pdf]

Significant Reduction of Systematic PAH and NPAH Discharges from Hazardous Waste Treatment by the Advanced Scrubbing and Circular GASMILD Combustion

Wei Tang[1,2], Yu-Lun Hsieh[2], Sheng-Lun Lin[2]*, Guor-Cheng Fang[3] Hyojun Lee[2,4]

[1] *School of Mechanical Engineering, Beijing Institute of Technology, Beijing 100081, China*

[2] *Department of Environmental Engineering, National Cheng Kung University, Tainan 70101, Taiwan*

[3] *Department of Safety, Health, and Environmental Engineering, Hungkuang University, Taichung 43302, Taiwan*

[4]*Department of Nuclear Engineering, Hanyang University, SeongDong-Gu, Seoul 04763, Korea*

*****Corresponding author: **Sheng-Lun Lin**. Phone: +886 6 275 7575. E-mail: z11208011@ncku.edu.tw or cbmsgml@gmail.com. Address: Department of Environmental Engineering, National Cheng Kung University, No.1, University Rd, East Dist, Tainan 70101, Taiwan.

Supplementary Material

Table S1 Properties of wastes, and the HAWTT system.

Properties and composition	Mean	RSD, %
Heat value, kcal/kg	5,359	23.4
Water contents, wt.%	43.3	44.6
Specific gravity	0.91	5.32
Chlorine [a], wt.%	21.8	75.03
Sulfur [a], mg/kg	21,923	20.3
Hg [a], ppm	427	34.2
Pb [a], ppm	22.2	21.7
Cd [a], ppm	743	48.5
Cr [a], ppm	515	18.1
As [a], ppm	2.84	46.3
Cu [a], ppm	584	36.1
Zn [a], ppm	1,703	41.5
Ag [a], ppm	136	31.1
Ni [a], ppm	497	24.1

[a] Analyzed by X-ray fluorescence every batch of waste; [b] Equivalence ratios of real to stoichiometric conditions in terms of fuel/air mass fractions

Table S2. Basic information and operating condition of HAWTTS units.

Parameters	Values
**Primary combustion chamber**	
Treatment capacities	
Organic waste, ton yr^{-1}	800
Inorganic waste, ton yr^{-1}	400
Combustion condition	$\Phi > 1.0$
Solid feeding rates, kg h^{-1}	100
Liquid feeding rate, kg h^{-1}	120
Recycled SFA, kg h^{-1}	24.6
Air feeding, Nm^3 mim^{-1}	1.0–1.2
Residual O_2, %	<3.0
In-chamber gas-phase temperature, °C	1,050–1,150
**Secondary combustion chamber**	
Combustion condition	$\Phi = 1.0–1.2$
Auxiliary diesel feeding, kg h^{-1}	0–60
Air feeding, Nm^3 mim^{-1}	6
Excess air, %	14.9–15.1
**Scrubber**	
Liquid flow rate (L/min)	211.9
pH	9.6
**Cyclonic demister**	
Flow velocity (m/s)	7-9
Flow temperature (℃)	60-70
Relative humidity (%)	100
**Baghouse**	
Area of fabric filter (m^2)	298
**Powder activated carbon**	
Charcoal diameter (μm)	<75
Moisture (%)	<3
Ash (%)	<18
pH	8–11
Apparent specific gravity (g/mL)	0.4–0.5

Φ: Equivalence ratios of real to stoichiometric conditions in terms of fuel/air mass fractions

Table S3 The primary information of PAH homologues.

PAH homologues	Abb.	TEF	NPAH homologues	Abb.	TEF
Naphthalene	NaP	0.001	1-Nitronaphthalene	1-NN	0.1
Acenaphthylene	AcPy	0.001	2-Nitronaphthalene	2-NN	-
Acenaphene	AcP	0.001	5-Nitroacenphthene	5-NA	-
Fluorene	Flu	0.001	2-Nitrofluorene	2-NF	0.01
Phenanthrene	PA	0.001	9-Nitroanthracene	9-NA	-
Anthracene	AnT	0.01	9-Nitrophenanthrene	9-NP	-
Fluoranthene	FL	0.001	3-Nitrophenanthrene	3-NP	-
Pyrene	Pyr	0.001	2-Nitrofluoranthene	2-NF	-
Benzo(a)anthracene	BaA	0.1	3-Nitrofluoranthene	3-NF	-
Chrysene	CHR	0.01	4-Nitropyrene	4-NP	0.1
Benzo(b)fluoranthene	BbF	0.1	1-Nitropyrene	1-NP	-
Benzo(k)fluoranthene	BkF	0.1	2-Nitropyrene	2-NP	-
Benzo(a)pyrene	BaP	1	7-Nitrobenzo(a)anthracene	7-NB	-
Indeno(1,2,3-cd)pyrene	IND	0.01	6-Nitrochrysene	6-NC	10
Dibenzo(a,h)anthracene	DBA	1			
Benzo(g,h,i)perylene	BghiP	0.1			

Table S4 Sampling locations and conditions within the HAWTTS facility.

Samp. Point	Sample Type	Location Description	Distance from Main Combustion Zone	Potential Sources of Interference
E	Feedstock (waste)	Waste feeding inlet to primary combustion chamber	0 m	None
A	Flue Gas (gas)	Outlet of secondary combustion chamber	Approx. XX m	Minimal
B	Flue Gas (gas)	Outlet of semi-dry cooling tower (SCB)	Approx. XX m	Condensate droplets
C	Flue Gas (gas)	Outlet of cyclone demister (CYCD)	Approx. XX m	Minimal
D	Flue Gas (gas)	Stack outlet (final discharge)	Approx. XX m	Atmospheric dilution
F	Residue (bottom ash)	Bottom ash collection pit of incinerator	Near 0 m	Ash resuspension
G	Residue (quenching tower ash)	Sludge collected from quenching water system	Approx. XX m	Water entrainment
H	Residue (SCB sludge)	Sludge collected from semi-dry cooling tower	Approx. XX m	Humidity variation
J	Residue (CYCD sludge)	Sludge collected from cyclone demister	Approx. XX m	Minimal
K	Residue (BH fly ash)	Fly ash collected from baghouse (BH)	Approx. XX m	Dust re-entrainment

Table S5 Computational fluid dynamics simulation analysis of operational conditions

**Air feeding $Nm^3\ mim^{-1}$**	**CO emission %**	**NO emission %**	**CO distribution**	**NO distribution**
0.6–0.8	1.25E-2	1.11E-4	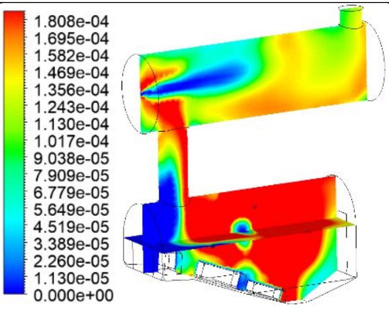	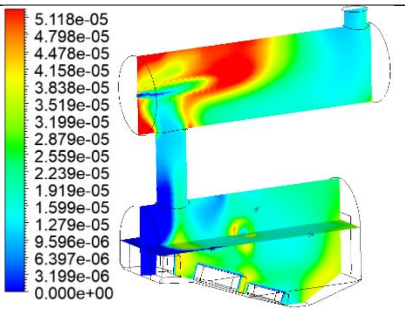
1.0–1.2	1.05E-2	1.02E-4	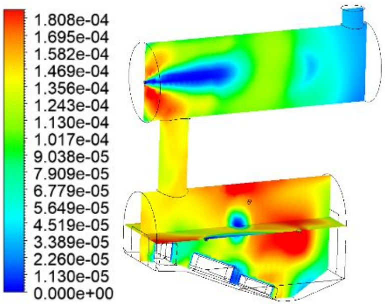	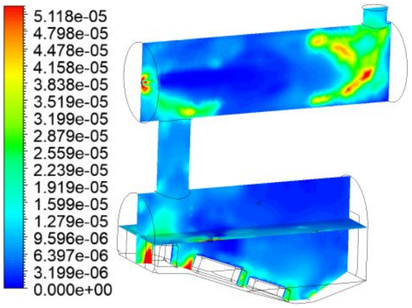
1.4–1.6	1.465E-2	1.73E-4	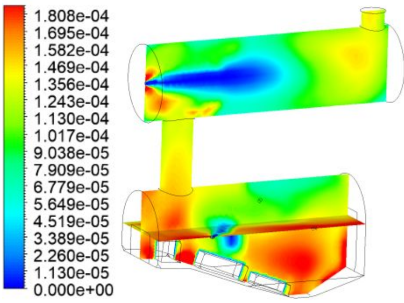	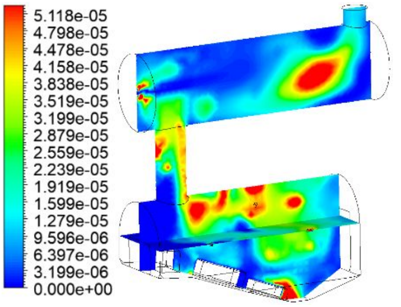

The simulation was modeled according to the actual dimensions and operating conditions of the incinerator. The turbulence model used was the standard k-epsilon model, while the radiation model employed was the Discrete Ordinates (DO) model. For species transport, the Eddy-Dissipation Concept (EDC) model was applied. This three-dimensional simulation determined the specific air feeding rate based on the NO and CO emissions at the outlet as performance indicators.

Analytical Methods and Quality Control for PAHs and NPAHs

Sampling and Extraction Procedures

PAH and NPAH sampling was conducted in accordance with the U.S. EPA Modified Methods 5 and 23. To separately capture particulate-phase and gas-phase species, isokinetic sampling was employed using quartz fiber filters and polyurethane foam (PUF) cartridges, respectively. Strict adherence to isokinetic conditions ensured the representativeness of size-segregated samples under varying flue gas flow conditions.

Particulate-phase PAHs and NPAHs were collected on pre-combusted quartz fiber filters (Whatman QM-A, 47 mm, baked at 450 °C for 6 hours). Gaseous-phase congeners were trapped using glass cartridges packed with pre-cleaned PUF plugs (Tisch Environmental, TE-1005). Sampling lines were leak-checked and pre-heated to avoid condensation of semi-volatile compounds.

Solid-phase samples were subjected to 16-hour Soxhlet extraction using a 1:1 (v/v) dichloromethane:n-hexane mixture. Liquid-phase PUF samples were extracted using ultrasonic agitation with the same solvent composition for 30 minutes. All extracts were then concentrated via rotary evaporation and purified through silica gel and activated alumina chromatography columns to remove polar interferences and enhance analytical resolution.

Instrumental Analysis

Quantification of PAHs and NPAHs was performed by gas chromatography-mass spectrometry (GC-MS, Agilent 6890N GC coupled with 5975B MSD) under selective ion monitoring (SIM) mode. The separation was achieved using a DB-5MS capillary column (30 m × 0.25 mm i.d., 0.25 μm film). The oven temperature program was optimized to resolve high molecular weight compounds: initial hold at 70 °C for 2 min, ramped to 150 °C at 25 °C/min, then to 280 °C at 8 °C/min with a final hold of 10 min. The injector operated in splitless mode with a helium carrier gas at a flow rate of 1.0 mL/min.

All samples were spiked with ^13C-labeled internal standards prior to extraction to monitor analyte recovery and ensure quantitative accuracy. Method performance was cross-validated using calibration curves constructed from certified standards and instrument blanks to check for carryover or memory effects.

Quality Control (QC) and Method Detection Limits (MDLs)

To ensure data integrity and analytical reproducibility, the following quality control procedures were implemented:

Pre-cleaning: All filters, PUFs, and glassware were cleaned using solvent extraction and high-temperature baking protocols to minimize background contamination.

Blanks and Duplicates: Field and procedural blanks were analyzed to detect contamination; duplicate samples were collected at representative locations to assess sampling reproducibility.

Calibration and Internal Standards: Multi-point calibration was performed for each PAH and NPAH congener, with daily performance verification using internal standard recoveries.

Storage: Extracts and sampling media were stored at −20 °C in amber containers to prevent photodegradation and volatilization prior to analysis.

Method detection limits (MDLs) were determined based on signal-to-noise ratios ≥3 in procedural blanks and were consistent with the Taiwan NIEA A801.90C standard. The full list of compound-specific MDLs, spike levels, and recovery rates is presented in Tables S6 and S7.

These procedures align with internationally recognized protocols and are designed to minimize sampling artifacts, ensure analytical precision, and enable congener-specific quantification of trace organic pollutants under complex flue gas conditions.

Table S6 Method detection limits and quality control parameters for PAHs.

**PAH Compounds**	**MDL, ng**	**QC spike, ng**	**QC recovery, %**
Napthalene	9.800	200	50~150
Acenaphthylene	0.594	200	50~150
Acenaphthene	0.224	200	50~150
Fluorene	0.439	200	50~150
Phenanthrene	0.901	200	50~150
Anthracene	0.203	200	50~150
Fluoranthene	0.547	200	50~150
Pyrene	0.650	200	50~150
Benzo[a]anthracene	0.253	200	50~150
Chrysene	0.354	200	50~150
Benzo[b]fluoranthene	0.208	200	50~150
Benzo[k]fluoranthene	0.207	200	50~150
Benzo[a]pyrene	0.372	200	50~150
Indeno(123-cd)pyrene	0.495	200	50~150
Dibenz[a,h]anthracene	0.413	200	50~150
Benzo[g,h,i]perylene	0.889	200	50~150

Table S7 Method detection limits and quality control parameters for NPAHs.

**NPAH Compounds**	**MDL, ng**	**QC spike, ng**	**QC recovery, %**
1-Nitronaphthalene	0.029	50	50~150
2-Nitronaphthalene	0.105	50	50~150
5-Nitroacenphthene	0.120	50	50~150
2-Nitrofluorene	0.067	50	50~150
9-Nitroanthracene	0.109	50	50~150
9-Nitrophenanthrene	0.372	50	50~150
3-Nitrophenanthrene	0.129	50	50~150
2-Nitrofluoranthene	0.389	50	50~150
3-Nitrofluoranthene	0.178	50	50~150
4-Nitropyrene	0.241	50	50~150
1-Nitropyrene	0.042	50	50~150
2-Nitropyrene	0.153	50	50~150
7-Nitrobenzo(a)anthracene	0.068	50	50~150
6-Nitrochrysene	0.093	50	50~150

Table S8 Mass flow rates and relative contributions of individual PAH congeners, LMW PAHs, and HMW PAHs in the SFA streams.

PAHs	Waste (ng/h)	SFA (ng/h)	Total Input (ng/h)
NaP	3250130288	1993734729	5243836000
AcPy	420720982	710095704	1130898944
AcP	31566672.8	4724811.53	36329504
Flu	76099047.2	32870484	108988512
PA	392086482	691795470	1083951648
AnT	14111081.6	34210947	48300544
FL	137812122	604607094	742412672
Pyr	256565120	918217155	1174827456
BaA	0	45517461	45489952
CHR	2393001.2	109801404	112215488
BbF	0	81709962	81715360
BkF	0	29373624	29355072
BaP	0	136843788	136782144
IND	0	139408152	139384544
DBA	0	3285206.72	3285207.3
BghiP	0	291754686	291781088
LMW PAHs	4184714553	3467432146	7652305152
HMW PAHs	396770243	2360518533	2757248983
LMW PAHs Ratio	0.913	0.595	0.735
HMW PAHs Ratio	0.086	0.405	0.265
